# Assessing the Impact of Data Preprocessing on Analyzing Next Generation Sequencing Data

**DOI:** 10.3389/fbioe.2020.00817

**Published:** 2020-07-30

**Authors:** Binsheng He, Rongrong Zhu, Huandong Yang, Qingqing Lu, Weiwei Wang, Lei Song, Xue Sun, Guandong Zhang, Shijun Li, Jialiang Yang, Geng Tian, Pingping Bing, Jidong Lang

**Affiliations:** ^1^Academician Workstation, Changsha Medical University, Changsha, China; ^2^Vascular Surgery Department, Tsinghua University Affiliated Beijing Tsinghua Changgung Hospital, Beijing, China; ^3^Department of Gastrointestinal Surgery, Yidu Central Hospital of Weifang, Weifang, China; ^4^Geneis Beijing Co., Ltd., Beijing, China; ^5^Department of Pathology, Chifeng Municipal Hospital, Chifeng, China

**Keywords:** the next generation sequencing, data preprocessing, mutation, cancer, HLA typing

## Abstract

Data quality control and preprocessing are often the first step in processing next-generation sequencing (NGS) data of tumors. Not only can it help us evaluate the quality of sequencing data, but it can also help us obtain high-quality data for downstream data analysis. However, by comparing data analysis results of preprocessing with Cutadapt, FastP, Trimmomatic, and raw sequencing data, we found that the frequency of mutation detection had some fluctuations and differences, and human leukocyte antigen (HLA) typing directly resulted in erroneous results. We think that our research had demonstrated the impact of data preprocessing steps on downstream data analysis results. We hope that it can promote the development or optimization of better data preprocessing methods, so that downstream information analysis can be more accurate.

## Introduction

In recent years, sequencing technologies, especially next-generation sequencing (NGS), have been widely used in scientific research and clinical applications. It allows for higher sequencing throughput and lower sequencing costs, and with the development and optimization of experimental and data analysis methods, the subsequent analysis results are increasingly accurate. For example, important techniques for detecting cancer-associated biomarkers using liquid biopsy techniques (Esposito et al., 2017) are essentially done using the NGS technology platform, especially in the detection of cell-free tumor DNA (ctDNA) in plasma, such as Duplex sequencing (Schmitt et al., 2012), Cancer Personalized Profiling by deep Sequencing (CAPP-Seq) (Newman et al., 2014), and Targeted Error Correction Sequencing (TEC-Seq) (Phallen et al., 2017). However, ctDNA sequencing data have strong background noise, contamination of sequencing adapters, unbalanced base distribution, sequencing quality and errors introduced during the experiments; these factors have a crucial impact on the accuracy of detecting low-frequency and even ultra-low-frequency mutations in ctDNA. Therefore, quality control and data preprocessing are especially important for obtaining downstream high-quality and high-confidence analytical data to reduce false positives and false negatives.

Illumina reads are commonly 36–300 nucleotide bases produced by a reversible-terminator cyclic reaction associated to base-specific colorimetric signals within the sequencing machine. Reads can be separated “single-end” or “paired-end” reads, in which case they are representing both extremities of the same nucleotide fragment. These colorimetric signals are translated into base calls by an internal Illumina software (CASAVA), represented in the FASTQ format (Cock et al., 2010), where each nucleotide is associated to an ASCII-encoded quality number corresponding to a PHRED score (Q) (Ewing and Green, 1998), which is in recent Illumina runs ranges from 0 to 41 and the error rate at each position ranges from 7.94e-5 to 1. Whatever the original cause of low quality or high error chance nucleotides, such as air bubbles, spot-specific signal noise, malfunctioning laser or lens, and so on, the *Q* value if encoded and stored together with the sequence information, and this confidence information can be used for subsequent analysis, together with the sequence information itself.

At present, there are many software programs for data quality preprocessing. Cutadapt (Martin, 2011), which is widely used, is the only stand-alone tool that can correctly trim color space reads. It can search for multiple adapters in a single run of the program and removes the best matching one. It can optionally search and remove an adapter multiple times, which is useful when (perhaps accidentally) library preparation has led to an adapter being appended multiple times. It can either trim or discard reads in which an adapter occurs. Reads that are outside a specified length range after trimming can also be discarded. In addition to adapter trimming, low-quality ends of reads can be trimmed using the same algorithm as Burrows-Wheeler Aligner (BWA). FastP (Chen et al., 2018b), as an all-in-one FASTQ preprocessor, provides functions including quality profiling, adapter trimming, read filtering, and base correction. It supports both single-end and paired-end short read data and provides basic support for long-read data, which are typically generated by PacBio and Nanopore sequencers. Trimmomatic (Bolger et al., 2014) includes a variety of processing steps for read trimming and filtering, but the main algorithmic innovations are related to the identification of adapter sequences and quality filtering. Trimmomatic uses a pipeline-based architecture, allowing individual “steps” (adapter removal, quality filtering, and so on) to be applied to each read/read pair in the order specified by the user. Each step can choose to work on the reads in isolation or work on the combined pair, as appropriate. The tool tracks read pairing and stores “paired” and “single” reads separately.

The data preprocessing software and algorithms have shown excellent results in published articles. We have also used them to obtain high-quality clean data to do downstream analysis, such as alignment and mutation detection. Generally, when there are false-positive or false-negative results, we tend to think this may be due to unreasonable parameter settings in the analysis process or other experimental reasons, but this may not always be the case. We analyzed and compared the raw sequencing data with commonly used data preprocessing software, such as Cutadapt, FastP, and Trimmomatic, and found that the data preprocessing results affected the subsequent detection results. Therefore, we realized that in the data preprocessing, we need to choose the software and algorithms carefully, and the data preprocessing algorithms need to be further improved according to actual data features. It is necessary to make different choices according to specific data analysis.

## Materials and Methods

### Sample Collection

HD753, a reference genomic DNA (gDNA), is used as the reference standard (Horizon Diagnostics^TM^, Waterbeach, United Kingdom) and contains 10 mutation variations: AKT serine/threonine kinase 1 (*AKT1*) p.E17K (5%), phosphatidylinositol-4,5-bisphosphate 3-kinase catalytic subunit alpha (*PIK3CA*) p.E545K (5.6%), epidermal growth factor receptor (*EGFR*) p.745-750del (5.3%), *EGFR* p.V769delinsVASV (5.6%), KRAS proto-oncogene, GTPase (*KRAS*) p.G13D (5.6%), notch receptor 1 (*NOTCH1*) p.P668S (5%), MET proto-oncogene, receptor tyrosine kinase (*MET*) p.V237fs (2.5%), BRCA2 DNA repair associated (*BRCA2*) p.A1689fs (5.6%), *EGFR* p.G719S (5.3%), B-Raf proto-oncogene, serine/threonine kinase (*BRAF*) p.V600E (18.2%), and *PIK3CA* p.H1047R (16.7%). The original HD753 reference has two replicates. We then used the standard sample to do three fivefold dilution experiments and every five-diluted sample has also two replicates, while the negative control sample, a healthy human white blood cells, also has two replicates.

All five human leukocyte antigen (HLA) typing samples and 75 mutation detection samples were obtained from lung cancer patients and informed written consent was obtained from the patients and de-identification. The 80 clinical samples we used were collected from October 2017 to May 2018.

### Experiment Workflow

gDNA for NGS-based mutation variations analysis was extracted using the GONOROAD Kit (Qiagen, Hilden, Germany) for formalin-fixed and paraffin-embedded (FFPE) tissue. DNA (200 ng) was used to build the library by using NEBNext Ultra II DNA library Prep Kit for Illumina (96 reactions) (NEB, Ipswich, MA, United States). Integrated DNA technologies (IDT, Skokie, IL, United States) customized probes were used for hybridization capture. We used the *Genesis* 41 gene tumor hotspot mutation customized panel (Supplementary Sheet 1) for eight gDNA standard samples and two negative control samples. Quantification was performed with a Library Quantification Kit – Illumina/Universal (Kapa Biosystems, Wilmington, MA, United States) on an ABI 7500 Real Time PCR system (Applied Biosystems, Waltham, MA, United States). A Quality control Agilent 2100 Bioanalyzer with a High Sensitivity DNA Kit was used for quality control (Agilent Technologies, Santa Clara, CA, United States). NGS analysis was performed on a Nextseq500 instrument according to the manufacturer’s instructions (Illumina, San Diego, CA, United States). With a NextSeq500/550 High Output V2 kit, Illumina Nextseq500 was used for DNA sequencing in 302 cycles, standing for paired-End 151bp.

The 75 clinical samples’ cell-free DNA was extracted using a QIAamp Circulating Nucleic Acid Kit (Qiagen, Hilden, Germany) according to the manufacturer’s instructions. The obtained DNA (20 ng/sample) was then used to build libraries using Accel-NGS^®^ 2S Plus DNA Library Kits (96 reactions; Swift Biosciences, Ann Arbor, MI, United States). Customized probes were obtained from Integrated DNA technologies (IDT, Skokie, IL, United States) and were used for hybridization capture. All cfDNA libraries utilized the *Genesis* 41 gene tumor hotspot mutation customized panel and were quantified using a Universal Library Quantification Kit (Kapa Biosystems, Wilmington, MA, United States) on an ABI 7500 Real-Time PCR system (Applied Biosystems, Waltham, MA, United States). Sample quality was evaluated using a high sensitivity DNA kit (Agilent Technologies, Santa Clara, CA, United States) with an Agilent 2100 Bioanalyzer per the manufacturer’s instructions. NGS with fusion detection was performed using a NextSeq 500/550 High Output v2 kit with a NextSeq 500 sequencer (Illumina, San Diego, CA, United States) for 302 cycles, with standing paired-end reads of 151 bp.

Five DNA samples for HLA typing analysis were extracted from the FFPE tumor tissues using the GeneRead DNA FFPE Kit (Qiagen, Hilden, Germany). DNA samples were normalized to yield a 100 − 250 ng input. Whole genome libraries were prepared using NEBNext^®^ Ultra^TM^ II DNA Library Prep (NEB, Ipswich, MA, United States) and through a series of steps including covaris shearing, end-repair, A-base addition, barcoded adapter ligation, and PCR amplification. Libraries were quantitated using a Qubit dsDNA HS Kit (Invitrogen, Carlsbad, CA, United States) and quality assessed with Agilent 2100 Bioanalyzer (Agilent Technologies, Santa Clara, CA, United States) as per the manufacturer’s protocol. Targeted enrichment was carried out on the prepared libraries to specifically pull down DNA fragments that contained the target site using custom 5′ biotinylated capture probes. Four libraries were then pooled at 125 ng each for a total of 500 ng. Cot-1 DNA (Sigma-Aldrich, MO, United States) and universal blocking oligonucleotides (IDT, Skokie, IL, United States) were added to the pooled libraries and dried in a SpeedVac. The dried mixture was then resuspended in IDT Hybridization Buffer and Hybridization enhancer (IDT, Skokie, IL, United States) and hybridized for 4 h with custom 5′ biotinylated capture IDT probes (IDT, Skokie, IL, United States) and BOKE probes (BOKE, Beijing, China). Streptavidin DynaBeads (Invitrogen, Carlsbad, CA, United States) were used for capture and washes were performed using xGenLockdown-Reagents Kit (IDT, Skokie, IL, United States). The final hybridized product was amplified using KAPA Hifi HotStart Ready Mix (Kapa Biosystems, Wilmington, MA, United States) and Illumina sequencing primers for a total of 15 cycles. Final target capture library quantification was performed using a Qubit dsDNA HS Kit (Invitrogen) and quality assessed with Agilent 2100 Bioanalyzer (Agilent Technologies, Santa Clara, CA, United States). With a NextSeq500/550 High Output V2 kit, Illumina Nextseq500 (Illumina) was used for DNA sequencing in 302 cycles, standing for paired-End 151 bp.

### Mutation Validation

*EGFR*-T790M (25), *EGFR*-L858R (26), *BRAF*-V600E (5), *PIK3CA*-E545K (6), *KRAS*-G12C (11), and *KRAS*-G12V (2) mutant allele frequencies were determined using a Digital Droplet PCR system (Bio-Rad Laboratories, Inc., Hercules, CA, United States), with a droplet size of 1 nL in a total reaction volume of 20 μL with ∼20 ng of cfDNA library utilized. All primers and probes were synthesized by IDT (Skokie, IL, United States). Droplet counts were determined using the QuantaSoft software (Bio-Rad) (Supplementary Sheet 7).

### HLA Typing Validation

Human leukocyte antigen typing was performed at the BFR Medical Laboratory (BFR, Beijing, China) by the high–resolution HLA sequence-based typing method (HLA-SBT).

### Data Analysis for Mutation Detection

**FIGURE 1:**
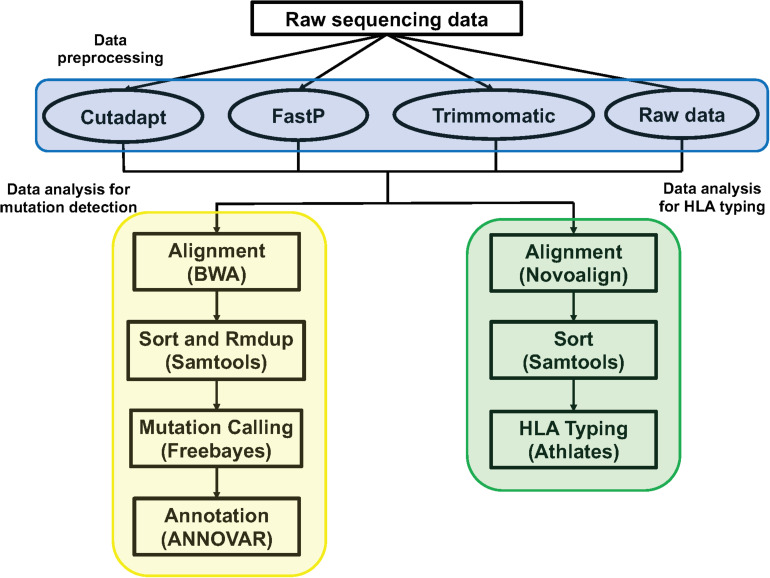
The pipeline of data analysis. The blue section was the three method of data preprocessing: Cutadapt, FastP, Trimmomatic, and raw sequencing data; the yellow section was the pipeline of data analysis for mutation detection; the green section was the pipeline of data analysis for HLA typing.

We used Cutadapt (version 1.3, *parameter: -b AGATCGGA AGAGCACACGTCTGAACTCCAGTCAC -b AGATCGGAAGAG CGTCGTGTAGGGAAAGAGTGTA -e 0.01 -m 15*), FastP (version 0.20.0, *parameter: -trim_poly_g*), and Trimmomatic (version 0.39, *parameter: PE -threads 4 -phred33 ILLUMINACLIP: TruSeq3-PE.fa:2:30:10 MINLEN:15*) to preprocess the raw sequencing data (*Fastq*), filtering out the adapter contamination reads, low-quality reads, and unpaired reads to get clean data. We used the Bwa aln (Version: 0.7.12-r1039) algorithm to align the clean data to the human reference genome (hg19) and get the Sequence Alignment/Map format (*sam)* file. For the Binary Alignment/Map format (*bam)* file, the *sam* file was sorted by samtools (Version: 0.1.19-44428cd). According to the bed interval file of the *Genesis* 41 gene tumor hotspot mutation customized panel, we used freebayes (version: v1.0.2-6-g3ce827d, *parameter: -j -m 10 -q 20 -F 0.001 -C 1 -t bed.file –f hg19.fa*) to call single nucleotide polymorphisms (SNPs) and insertions or deletions (indels), and then used ANNOVAR to do the annotation (Figure 1).

### Data Analysis for HLA Typing

The method and parameters of data preprocessing were consistent with the above. We used Novoalign (version: V3.09.02, parameter: -t 30 -o SAM -r all -l 80 -e 100 -i PE 200 140) to align the clean data to the HLA reference sequence. We then used samtools (version: 1.3.1) to sort the *sam* files to get the *bam* files. We used Athlates (version: 1.0, default parameter) for typing analysis of HLA-A^∗^, HLA-B^∗^, and HLA-C^∗^ (Figure 1).

## Results

### No Significant Difference in the Impact on Data Quality After Data Preprocessing

**FIGURE 2:**
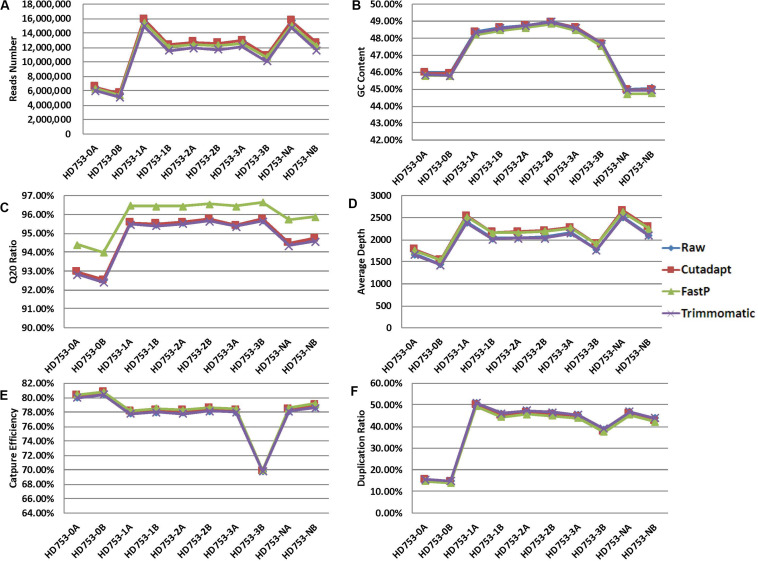
Quality control statistical distribution of Cutadapt, FastP, and Trimmomatic preprocessed data and raw sequencing data. **(A)** Statistical distribution of the number of reads, **(B)** statistical distribution of the GC content, **(C)** statistical distribution of the Q20 ratio, **(D)** statistical distribution of the average depth, **(E)** statistical distribution of the capture efficiency, and **(F)** statistical distribution of the duplication rate.

For the 10 standard samples data, calculating the number of reads, GC content, Q20 ratio, average depth, capture efficiency, and duplication rate after data preprocessing (Figures 2A–F and Supplementary Sheet 2), we found that the data of the three software-processed indicators, except the Q20 ratio, showed no significant difference (the *p*-value of the two-tailed heteroscedastic *T*-test was > 0.05). The Q20 ratio after FastP treatment was significantly improved, and the two-tailed heteroscedastic *T*-test *p*-values were 0.036 (vs. Raw data), 0.040 (vs. Cutadapt), and 0.026 (vs. Trimmomatic).

For the 75 clinical samples data, we also found the same conclusion that the data, except the Q20 ratio, showed no significant difference (Supplementary Figure 1 and Supplementary Sheet 6). The Q20 ratio after FastP treatment was significantly improved, and the two-tailed heteroscedastic *T-*test *p*-values were 1.69476E-10 (vs. Raw data), 3.05502E-10 (vs. Cutadapt), and 2.24745E-11 (vs. Trimmomatic).

### Frequency of Mutations Detected After Data Preprocessing May Be Affected

**FIGURE 3:**
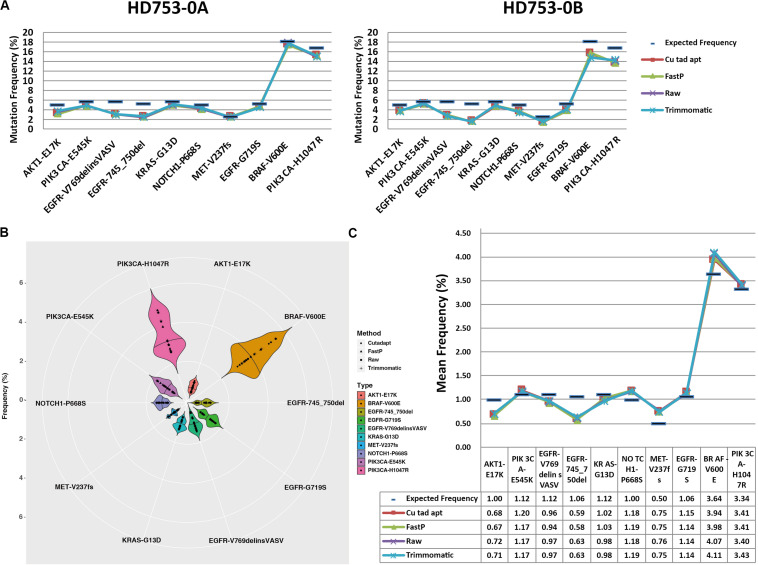
Distribution of hotspot mutation detection in the reference standard samples: **(A)** frequency distribution of the hotspot mutation detection in the two experimental replicates of the reference standard samples, **(B)** frequency distribution of the hotspot mutation detection in each experimental repeat of the fivefold dilution standard samples, and **(C)** average frequency distribution of the hotspot mutation detection in each experimental repeat of the fivefold dilution standard samples.

For the 10 standard samples data, we found that all of the hotspot mutations were detected in raw data, Cutadapt, FastP, and Trimmomatic preprocessing data in the two replicate reference standard gDNAs and the fivefold diluted HD753 specimens (Supplementary Sheet 3), while false positive results of *EGFR* p.G719S were found in all the negative control samples (HD753-NB). It may have been caused by sequencing errors or contamination introduced during the experiment. We found that the four preprocessing data analysis results had lower mutation frequencies than the expected frequencies of HD753-0A and HD753-0B (Figure 3A), which may be related to the experimental capture operation. There was no statistical difference between the distribution of frequencies of the four data types (the *p*-values of the two-tailed heteroscedastic *T*-test were > 0.05). But for the repeated dilution samples, the detected mutation frequency fluctuated greatly (Figures 3B,C). We assumed that a mutation frequency greater than 1% was used as a threshold for positive result for the FFPE or tissue samples. For a hotspot mutation *AKT1* p.E17K in HD753-1A, the detection results after Cutadapt and FastP data pretreatment were positive, and the detection frequencies were 1.06% (41/3869) and 1.00% (38/3785), respectively. Meanwhile, the raw data and Trimmomatic treatments were negative, with detection frequencies of 0.95% (34/3579) and 0.96% (34/3549), respectively. For *EGFR* p. 745_750del of HD753-2A, the results were negative after pretreatment with Cutadapt and FastP data, and the detection frequencies were 0.97% (20/2055) and 0.98% (20/2051), respectively. The results of the raw data and Trimmomatic treatment were positive, and the detection frequencies were 1.05% (20/1900) and 1.06% (20/1889), respectively. While the mutation of *NOTCH1* p.P668S in HD753-2A was detected as a positive result, which was preprocessed by FastP data, the detection frequency was 1.12% (32/2860). The data preprocessed by Cutadapt, Trimmomatic, and the raw data were negative, and the detection frequency was 0.98% (28/2857), 0.98% (26/2659), and 0.97% (26/2686), respectively. We also found that the results of Trimmomatic data pretreatment were basically consistent with the raw sequencing data, and the results of Cutadapt and FastP data pretreatment were consistent. It showed that there were no significant differences in the four methods of mutation support reads number. However, the sequencing depth of the mutation sites were quite different. That may be because Cutadapt and FastP only trim the reads, making the sequence shorter and achieving multi-alignment in the alignment process, which could be increasing the probability of the alignment. In contrast, in our paired-end preprocessing data, Trimmomatic retained the sequenced full-length sequence to be consistent with the raw sequencing data (Supplementary Sheet 3).

For the 75 clinical samples data, we used digital droplet PCR system to determine the mutations’ frequency, and the frequency limit was 0.1%. Consistent with the results found in the results of the standard, there was some fluctuation in the detection frequency after 4 data preprocessing method, but the coefficient of determination *R*^2^ of raw data, Cutadapt, Fastp, and Trimmomatic was 0.9386, 0.9381, 0.9416, and 0.9416, respectively. Compared with the result of ddPCR, the detection rate of data preprocessing by raw data, Cutadapt, Fastp, and Trimmomatic was 94.67% (71/75), 100% (75/75), 98.67% (74/75), and 96.00% (72/75), respectively (Supplementary Sheet 7). We found that the false negatives had a low mutation frequency (Table 1), and the effect of Cutadapt was the best compared to the other three methods.

**TABLE 1:** Compared with ddPCR results, the false negative results of data preprocessing by raw data, Cutadapt, Fastp, and Trimmomatic.

**Sample_ID**	**Mutation_Type**	**ddPCR (%)**	**Raw_data (%)**	**Cutadapt (%)**	**Fastp (%)**	**Trimmomatic (%)**
T790M-sample21	EGFR:p.T790M	0.17	0.00	0.14	0.00	0.00
L858R-sample19	EGFR:p.L858R	0.32	0.00	0.15	0.15	0.15
V600E-sample8	BRAF:p.V600E	0.31	0.00	0.26	0.26	0.00
E545K-sample10	PIK3CA:p.E545K	0.24	0.00	0.16	0.14	0.00

*Mutations’ frequency limit was 0.1%.

### HLA Typing Data After Preprocessing Had a Significant Impact on Data Quality

**FIGURE 4:**
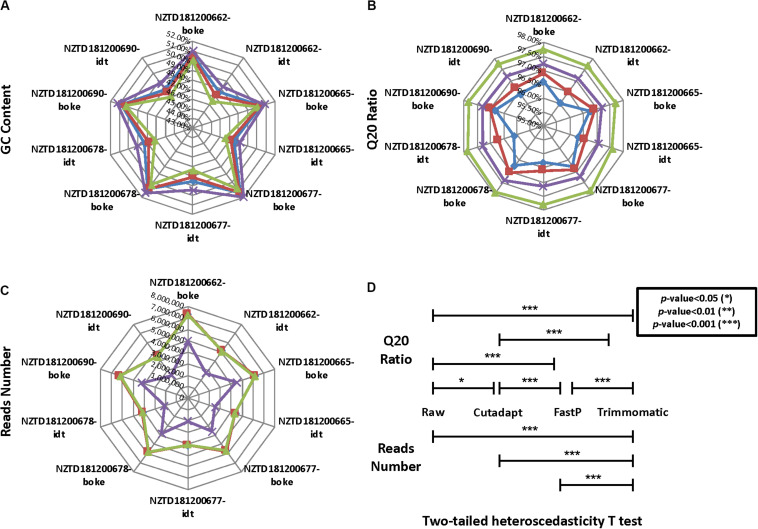
Quality control statistical distribution of Cutadapt, FastP, Trimmomatic preprocessed data, and raw sequencing data for 10 HLA typing samples. **(A)** Statistical distribution of the GC content, **(B)** statistical distribution of the Q20 ratio, **(C)** statistical distribution of the reads number, and **(D)** significant level for the four data types in the Q20 ratio and the reads number.

We calculated the reads number, GC content, and Q20 ratio for 10 HLA typing samples after data preprocessing (Figures 4A–C and Supplementary Sheet 4). We found that the data distribution after the three software treatments had significant fluctuations. The Q20 ratio was statistically significant based on the two-tailed heteroscedasticity test. The number of reads after Trimmomatic data preprocessing was significantly different from the distribution of the remaining three data types (Figure 4D).

### Data Preprocessing May Affect HLA Typing Analysis

We performed HLA typing data analysis on five samples captured by BOKE and IDT probes. We obtained incorrect typing results with the data after pretreatment of Cutadapt and FastP (Tables 2a, 2b and Supplementary Sheet 5). After pretreatment with Cutadapt and FastP data, the sample NZTD181200662 showed errors in the typing analysis of HLA-A and HLA-C, whether it was the BOKE probe capture or IDT probe capture, which was inconsistent with the validation results (such as Table 1, the red background was shown). The results of the raw data and Trimmomatic data preprocessing were consistent with the validation results. Since the NZTD181200690 sample was classified incorrectly in the four analysis results, it may have been caused by experiments or sequencing errors. Therefore, for the overall result of the BOKE probe capture, the accuracy after treatment with Cutadapt, FastP, raw data, and Trimmomatic was 86.67, 80.00, 93.33, and 93.33%, respectively. For the overall results of the IDT probe capture, the accuracy rates after treatment with Cutadapt, FastP, raw data, and Trimmomatic were 86.67, 86.67, 93.33, and 93.33%, respectively.

**TABLE 2a:** Summary of HLA typing results in the four data types with BOKE capture probes.

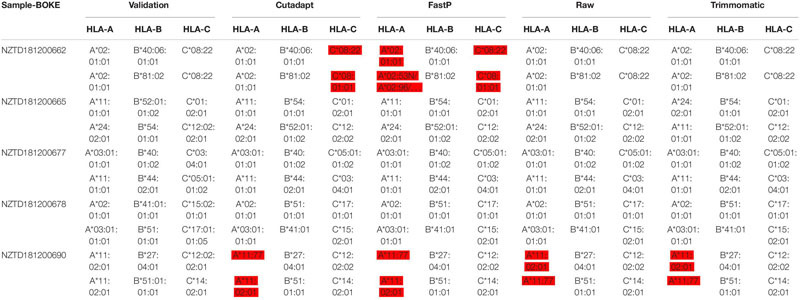

*The color values represented that the predicted results were inconsistent with the validated results.*

**TABLE 2b:** Summary of HLA typing results in the four data types with IDT capture probes.

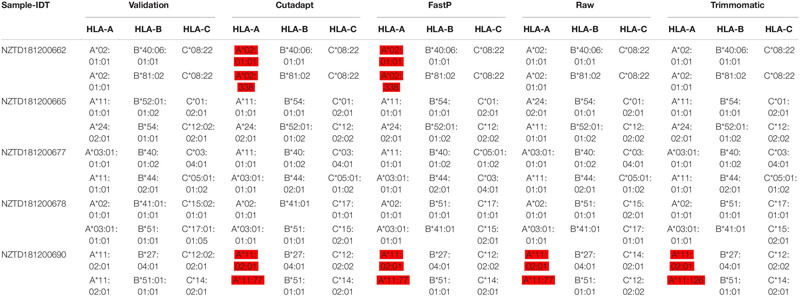

*The color values represented that the predicted results were inconsistent with the validated results.*

**FIGURE 5:**
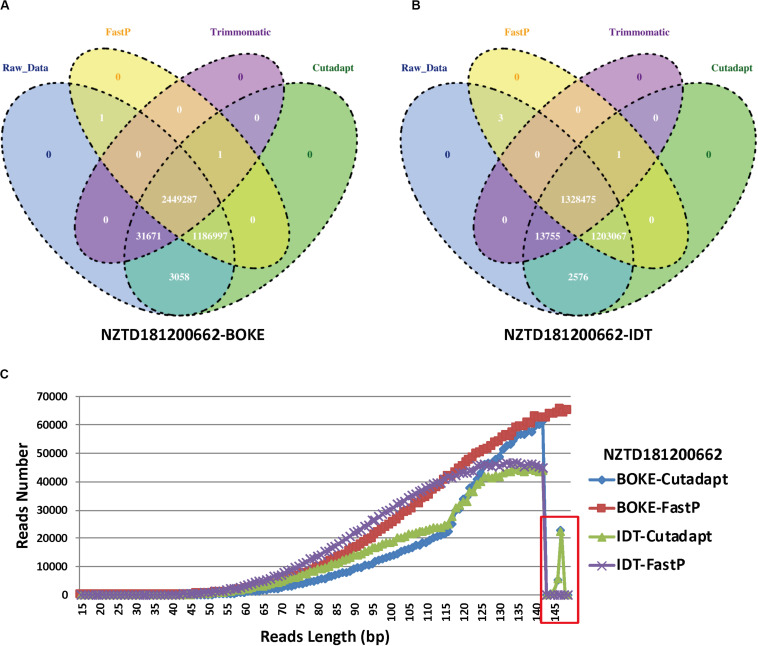
Statistics for the reads ID in the four data preprocessing types of sample NZTD181200662. **(A)** Four kinds of reads ID difference by BOKE probe capture data processing, **(B)** four kinds of reads ID difference by IDT probe capture data processing, and **(C)** the length distribution in the Cutadapt preprocessing data for the BOKE and IDT probes and the FastP preprocessing data for the BOKE and IDT probes.

For the NZTD181200662 sample, we extracted the reads ID of the sequencing data captured by the BOKE probe capture and the IDT probe (Figures 5A,B), and we wanted to know if the sequence reads causing the typing error had certain characteristics. We found that the three preprocessed data were highly consistent with the raw sequencing data, but the Trimmomatic preprocessed data also had many specific reads, accounting for 32.64 and 47.52% of the FastP preprocessing data captured by the BOKE and IDT probes, respectively. This phenomenon was basically the same in the remaining samples (Supplementary Figures 3–6). Due to the high accuracy of the raw data and Trimmomatic preprocessed data, we assumed that the read features that caused the incorrect HLA typing data of the Cutadapt and FastP data were in their specific reads compared with the raw data and Trimmomatic data. We extracted this part of the read and analyzed the length distribution of the reads. We found that the length of the read from 143 bp to 149 bp was significantly reduced (Figure 5C). Therefore, we extracted the 143–149 bp reads from the NZTD181200662’s BOKE and IDT probes captured data processed by Cutadapt and FastP for HLA-A and HLA-C typing, respectively. The results were consistent with the validation results for the BOKE’s capture probes. The HLA-A typing results of Cutadapt and FastP processed data were A^∗^02:01:01/A^∗^02:01:01 and the HLA-C typing results were C^∗^08:22/C^∗^08:22. For the IDT’s capture probe, the HLA-A typing results of Cutadapt and FastP processed data were A^∗^02:01:01/A^∗^02:01:01 and the HLA-C typing results were C^∗^08:22/C^∗^08:22.

## Discussion

Data quality control and preprocessing play an important role in data analysis in scientific research and clinical fields, and it is often the first step in data analysis. We believe that it can help us evaluate the experimental steps or problems in the sequencing process, and also reduce the sequence of low-quality or adapter contamination, reduce the computational cost, and allow us to obtain high-quality sequencing sequences for downstream analysis, making the analysis results more reliable. When false-positive or false-negative results are obtained, we usually think it is caused by (i) experimental factors, such as errors introduced by PCR or sample contamination; (ii) sequencing factors, such as sequencing quality and data contamination caused by index hopping when splitting data; or (iii) analysis software parameters setting factors, such as alignment software or specific parameter adjustment of downstream personalized analysis. There have been some studies that have done some comparisons of data preprocessing methods, for example, Del Fabbro et al. (2013) evaluated nine different trimming algorithms in four datasets and three common NGS-based applications (RNA-Seq, SNP calling, and genome assembly) (Chen et al., 2018a). But until now, we still did not notice that the data preprocessing step may also have a certain impact on the analysis results. We may even consider the notion that the sole purpose of data preprocessing is to reduce the downstream computing consumption and describe the quality of the sequencing data, as the actual importance and meaning have been previously neglected.

In this study, we compared commonly used data preprocessing software and found differences in the detection of hotspot mutations and HLA typing. Although the detection results may be affected by the three factors described above, for the different processing of the same data and the subsequent set of analysis processes, this could reflect the difference between the different pretreatment methods and the impact on the detection results. For the current “liquid biopsy” method, the sample testing requirements are to detect ctDNA mutations in the plasma to guide subsequent targeted drug therapy or real-time monitoring, but the ctDNA’s content in the plasma is very small (Bettegowda et al., 2014). For the accuracy of detecting mutations, each step in the experiment and analysis process should require strict quality control. Each step plays an important role in the detection results and cannot be ignored. Particularly for the detection of low-frequency or ultra-low-frequency mutations such as hotspot mutations, we showed that if the sequencing depth and mutation support reads number changes, it may directly lead to false-positive or false-negative results, which has a huge impact on clinical testing.

Currently, there are many available data quality control and preprocessing software programs, in addition to the three methods described in the article, such as FASTQC (Andrews, 2010), SOAPnuke (Chen et al., 2018c), and NGSQC (Dai et al., 2010). But most methods for the strategy of data preprocessing are to cut off all subsequent bases as long as the average quality of the bases in a certain bin or consecutive bases is below a certain threshold to reduce memory consumption and I/O reading, increasing the speed of operation. They do not notice the distribution of the actual low-mass bases in the sequence, which could result in many short sequences and may reduce the accuracy of downstream alignment and increase the sequencing depth of some reference sites. Thus, the analysis results may be inaccurate, and the effect may not be as good as the result of not doing data preprocessing, which was also confirmed in our analysis results. As the sequencing throughput becomes higher and higher, the sequencing read length becomes longer and longer, but the longer the sequencing read length, the worse the sequencing quality. Therefore, data preprocessing becomes increasingly important in data analysis. Existing principles and methods of data preprocessing for the long sequencing read length are worth considering. Our research explains the impact of data preprocessing steps on downstream data analysis results. We hope that our study can promote the development or optimization for the data preprocessing methods, so that downstream information analysis can be more accurate.

## Data Availability Statement

The standard sample and 5 HLA-typing clinical sample FASTQ data files are available from the NCBI Sequence Read Archive (SRA) database (BioProject ID: PRJNA556054). The 75 clinical sample FASTQ data files are available from the NCBI Sequence Read Archive (SRA) database (BioProject ID: PRJNA562379).

## Author Contributions

JL, BH, PB, and GT designed the study, collected, analyzed, and interpreted the data, and wrote the manuscript. HY, JY, QL, WW, LS, XS, GZ, and RZ did the experiment. RZ and SL reviewed the manuscript. All authors approved the final version of the manuscript.

## Conflict of Interest

JY, QL, WW, LS, XS, GZ, GT, and JL were employed by the company Geneis Beijing Co., Ltd.

The remaining authors declare that the research was conducted in the absence of any commercial or financial relationships that could be construed as a potential conflict of interest.
