# Supplementary material for: Assessing the Impact of Data Preprocessing on Analyzing Next Generation Sequencing Data
Source: Front Bioeng Biotechnol. 2020 Jul 30;8:817. doi: 10.3389/fbioe.2020.00817 (PMC7409520; doi:10.3389/fbioe.2020.00817)
Supplement: Supplementary file 1 [file Data_Sheet_1.pdf]

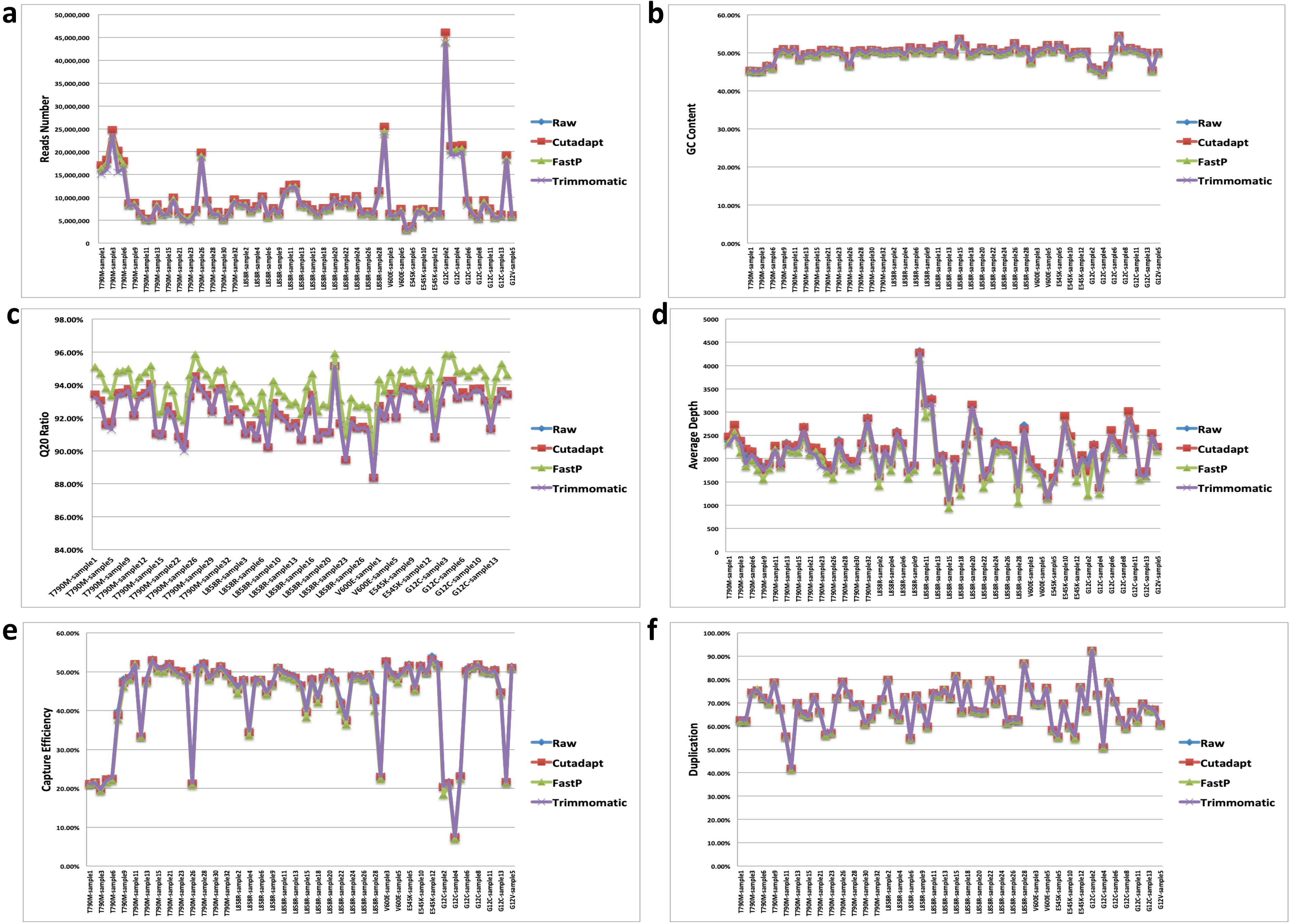

Supplementary Figure 1. Quality control statistical distribution of Cutadapt, FastP and Trimmomatic preprocessed data and raw sequencing data. (a) Statistical distribution of the number of reads, (b) statistical distribution of the GC content, (c) statistical distribution of the Q20 ratio, (d) statistical distribution of the average depth, (e) statistical distribution of the capture efficiency, and (f) statistical distribution of the duplication rate.

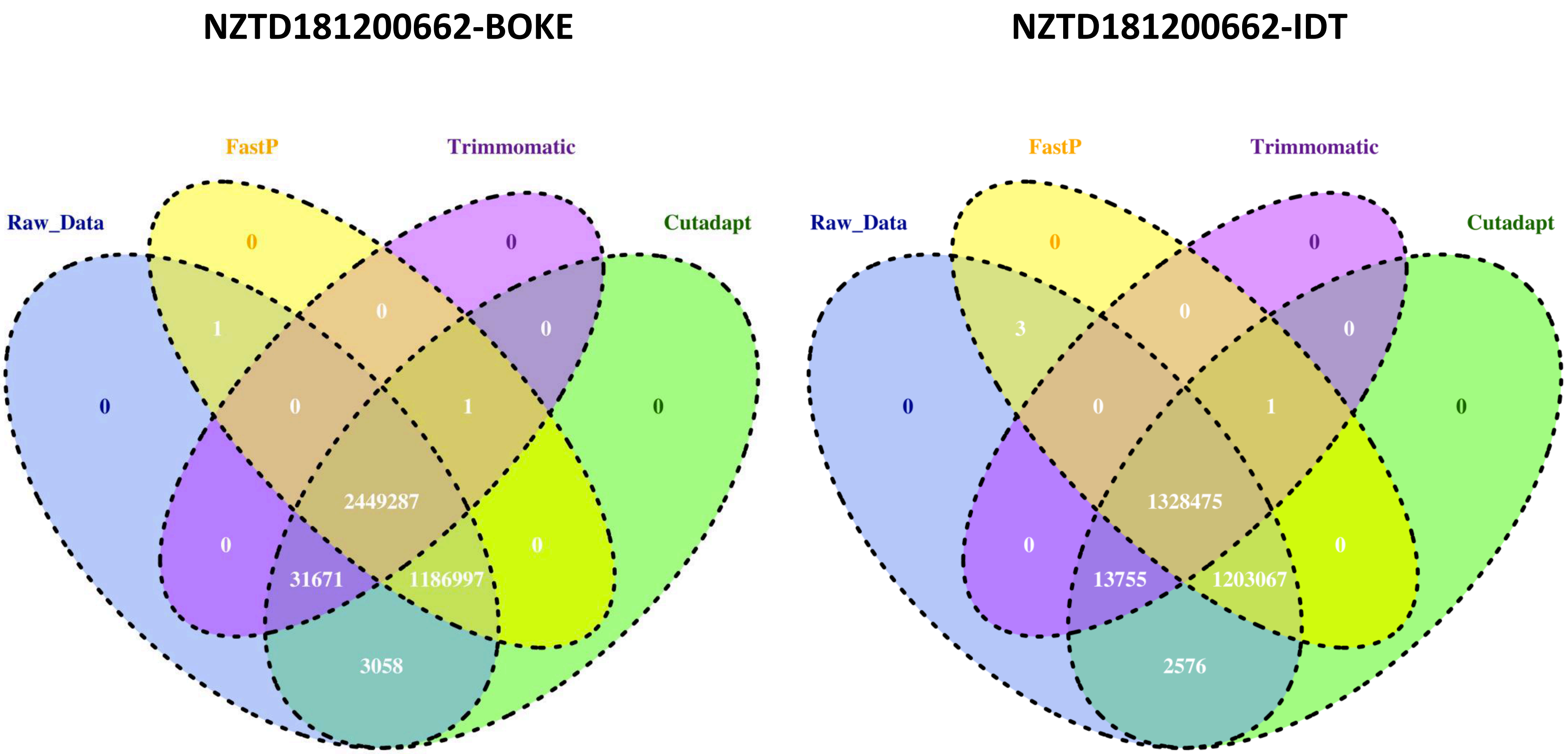

Supplementary Figure 2. Statistics for the reads ID in the four data preprocessing types of sample NZTD181200662. (a) four kinds of reads ID difference by BOKE probe capture data processing, (b) four kinds of reads ID difference by IDT probe capture data processing,

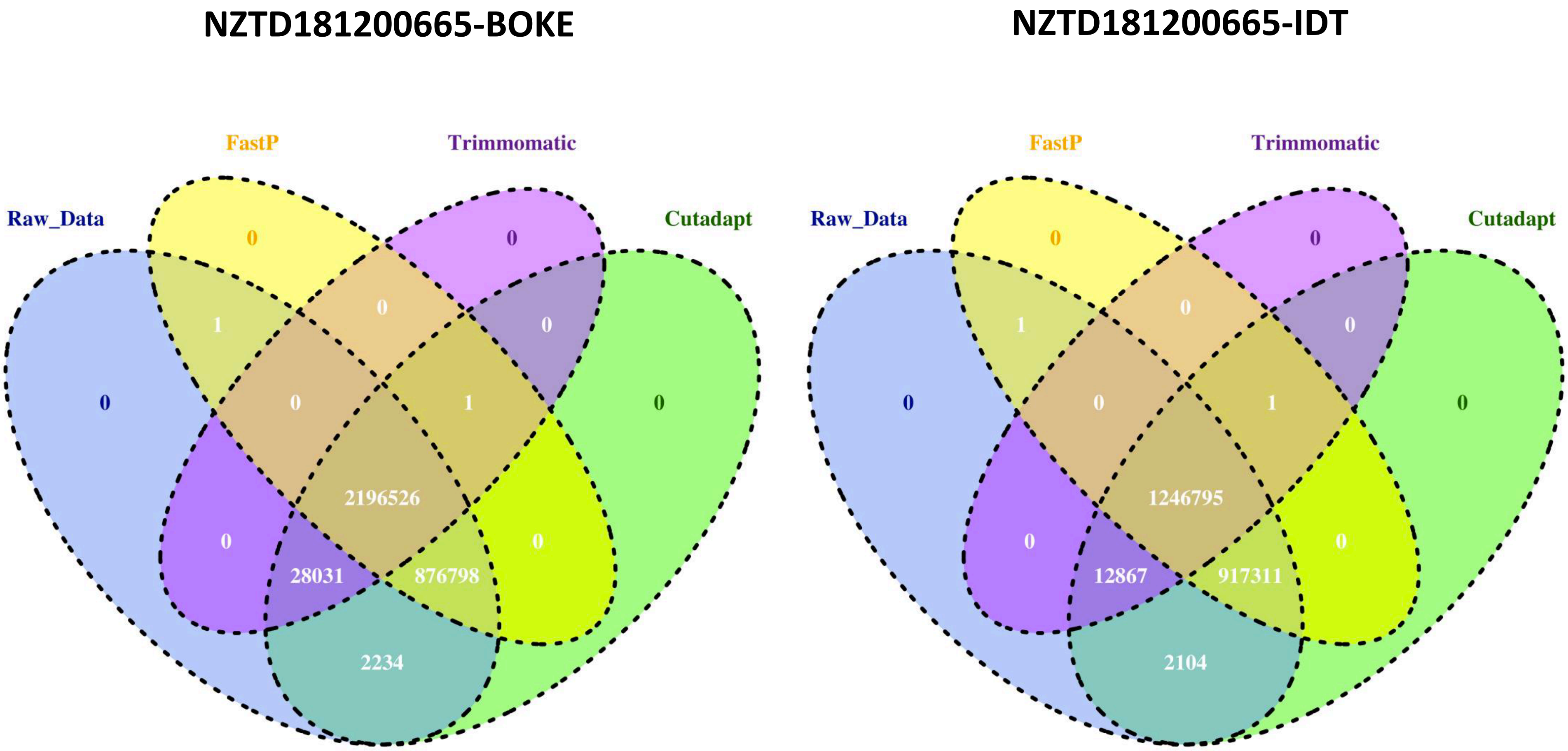

Supplementary Figure 3. Statistics for the reads ID in the four data preprocessing types of sample NZTD181200665. (a) four kinds of reads ID difference by BOKE probe capture data processing, (b) four kinds of reads ID difference by IDT probe capture data processing,

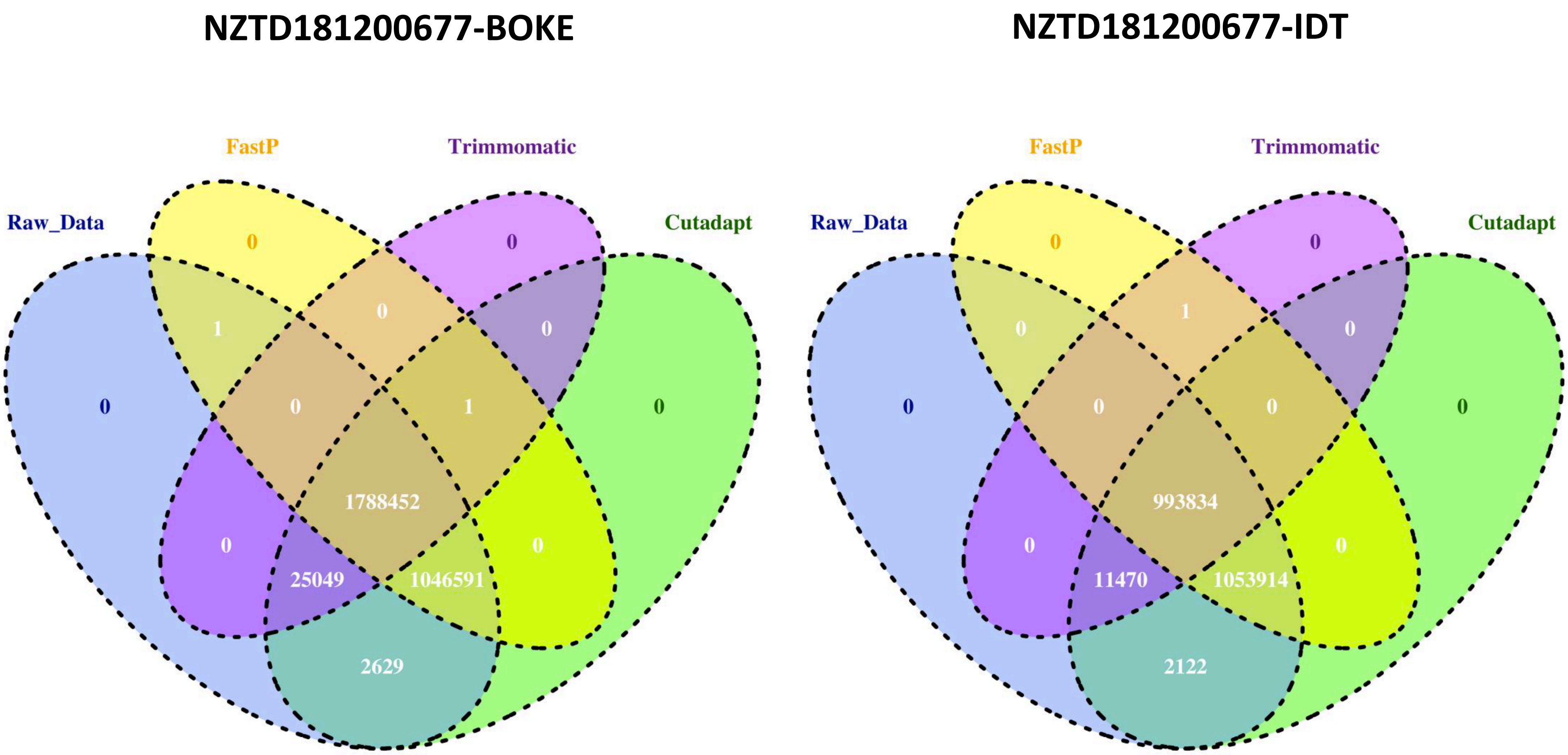

Supplementary Figure 4. Statistics for the reads ID in the four data preprocessing types of sample NZTD181200677. (a) four kinds of reads ID difference by BOKE probe capture data processing, (b) four kinds of reads ID difference by IDT probe capture data processing,

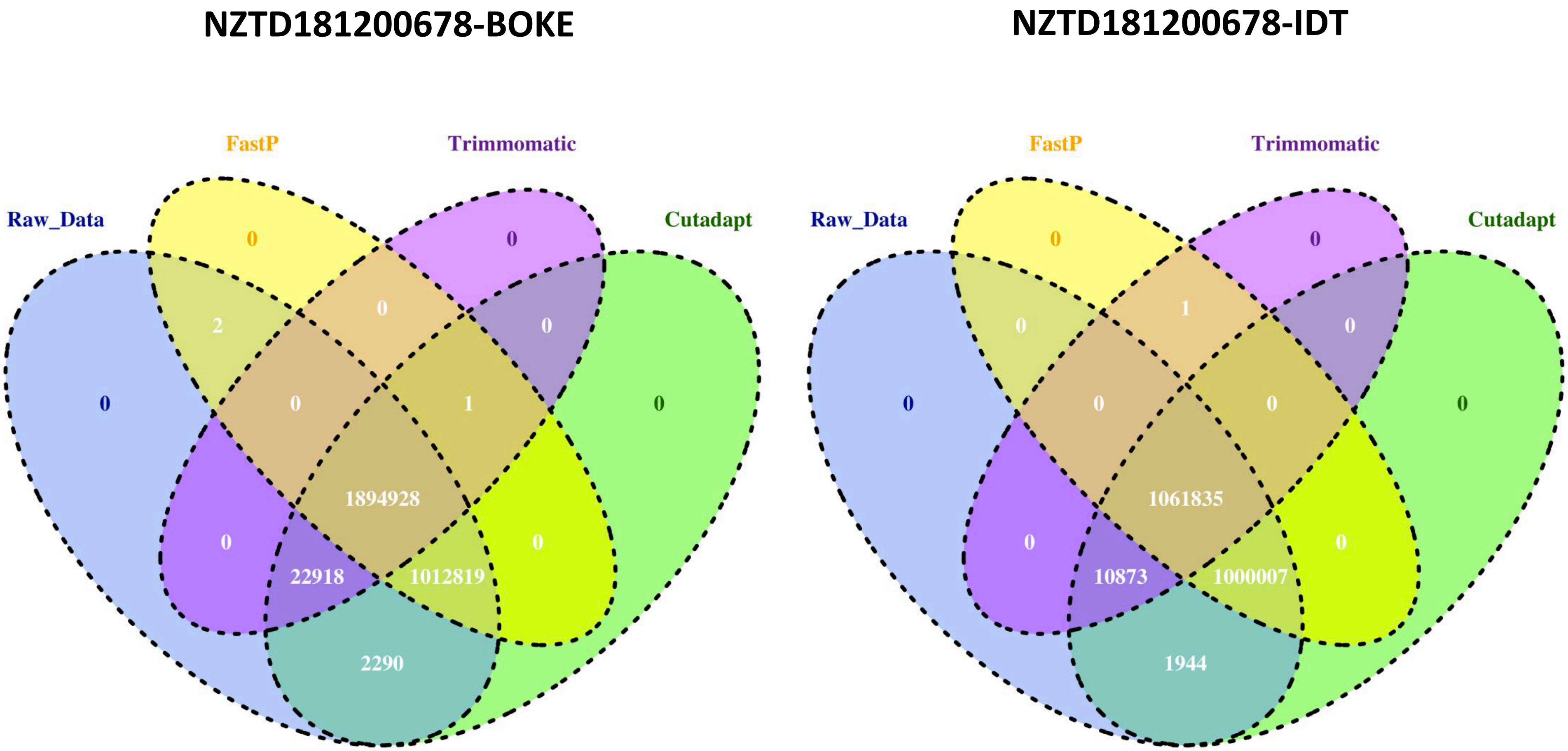

Supplementary Figure 5. Statistics for the reads ID in the four data preprocessing types of sample NZTD181200678. (a) four kinds of reads ID difference by BOKE probe capture data processing, (b) four kinds of reads ID difference by IDT probe capture data processing,

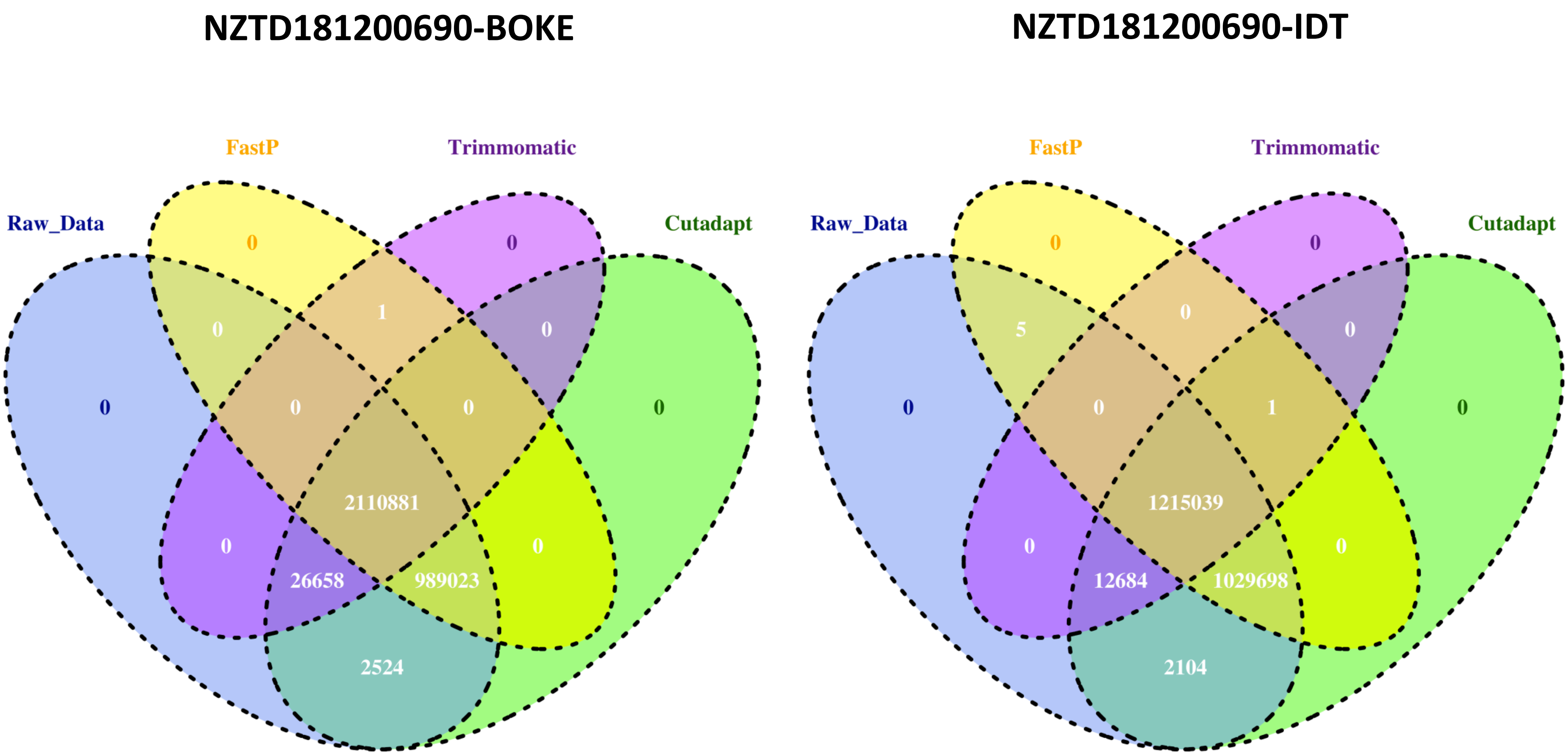

Supplementary Figure 6. Statistics for the reads ID in the four data preprocessing types of sample NZTD181200690. (a) four kinds of reads ID difference by BOKE probe capture data processing, (b) four kinds of reads ID difference by IDT probe capture data processing,
